# *Numt*-Mediated Double-Strand Break Repair Mitigates Deletions during Primate Genome Evolution

**DOI:** 10.1371/journal.pgen.1000237

**Published:** 2008-10-24

**Authors:** Einat Hazkani-Covo, Shay Covo

**Affiliations:** 1 National Evolutionary Synthesis Center, Durham, North Carolina, United States of America; 2 Laboratory of Molecular Genetics, Chromosome Stability Section, National Institute of Environmental Health Sciences, National Institutes of Health, Research Triangle Park, North Carolina, United States of Amercia; Stanford University, United States of America

## Abstract

Non-homologous end joining (NHEJ) is the major mechanism of double-strand break repair (DSBR) in mammalian cells. NHEJ has traditionally been inferred from experimental systems involving induced double strand breaks (DSBs). Whether or not the spectrum of repair events observed in experimental NHEJ reflects the repair of natural breaks by NHEJ during chromosomal evolution is an unresolved issue. In primate phylogeny, nuclear DNA sequences of mitochondrial origin, *numts*, are inserted into naturally occurring chromosomal breaks via NHEJ. Thus, *numt* integration sites harbor evidence for the mechanisms that act on the genome over evolutionary timescales. We have identified 35 and 55 lineage-specific *numts* in the human and chimpanzee genomes, respectively, using the rhesus monkey genome as an outgroup. One hundred and fifty two *numt*-chromosome fusion points were classified based on their repair patterns. Repair involving microhomology and repair leading to nucleotide additions were detected. These repair patterns are within the experimentally determined spectrum of classical NHEJ, suggesting that information from experimental systems is representative of broader genetic loci and end configurations. However, in incompatible DSBR events, small deletions always occur, whereas in 54% of *numt* integration events examined, no deletions were detected. *Numts* show a statistically significant reduction in deletion frequency, even in comparison to DSBR involving filler DNA. Therefore, *numts* show a unique mechanism of integration via NHEJ. Since the deletion frequency during *numt* insertion is low, native overhangs of chromosome breaks are preserved, allowing us to determine that 24% of the analyzed breaks are cohesive with overhangs of up to 11 bases. These data represent, to the best of our knowledge, the most comprehensive description of the structure of naturally occurring DSBs. We suggest a model in which the sealing of DSBs by *numts*, and probably by other filler DNA, prevents nuclear processing of DSBs that could result in deleterious repair.

## Author Summary

Changes to DNA sequence are the major source of variation in evolution. Those changes often arise from damage to DNA that is repaired in a way that fails to restore the original sequence. One type of DNA damage is a chromosomal double-strand break. Such breaks are mostly studied experimentally in model systems, because naturally occurring chromosomal breaks are hard to follow. Here, we used an evolutionary approach to study the repair of naturally occurring chromosomal breaks. Throughout evolutionary history, fragments of the mitochondrial genome, known as *numts* (nuclear sequences of mitochondrial origin), have been inserted into the nuclear genome. *Numts* are passively captured into random chromosomal breaks, leaving sequence traces in genomes. Humans and chimpanzees share a recent common ancestor and their genomes share high sequence similarity; therefore, their species-specific *numts* can be used to follow both some of the break structure and repair mechanisms. Comparing naturally occurring break and repair patterns with experimental repair patterns identified similarities but also highlighted a clear difference. Experimental breaks usually involve deletions, while deletions were significantly less frequent in the *numt* based repair system. We propose that extra-chromosomal DNA sequences, like *numts*, play a role in maintaining genome integrity by protecting naturally occurring chromosomal breaks from further deleterious processing.

## Introduction

The major mechanism of double-strand break repair (DSBR) in mammalian cells involves the religation of the two broken ends of the damaged chromosome by DNA Ligase IV, a process known as classical non-homologous end joining (NHEJ) [1],[2]. The hallmarks of NHEJ activity are, first, the tendency of the DNA termini to form base-pair complements, also known as microhomology-mediated repair, and second, that it is an error prone process in which nucleotides are often deleted or added. The mechanisms of chromosomal NHEJ repair of double strand breaks (DSBs) have been studied in great detail in two experimental model systems: V(D)J recombination [2],[3] and I-SceI-induced DSBs [4]–[7]. In each case, the repair of specific DSB end configurations generated by endonucleases at specific loci has been studied. It is unclear if the same repair pattern is shared between experimental and naturally occurring breaks, as the latter are much more diverse in respect to their genomic locations and break configurations [8],[9]. Inaccurate repair of naturally occurring breaks has driven chromosome evolution by introducing structural changes [10]. Whether or not the spectrum of NHEJ repair events observed in experimental systems is a reflection of repair of DSBs during chromosomal evolution is an unresolved issue.

Evidence has accumulated to suggest that extra-chromosomal DNA (also known as filler DNA) is captured into DSB repair sites via NHEJ [5], [11]–[17]. If true, then evidence for DSBR should be preserved in genomes and identifiable in genome comparisons spanning short evolutionary times. Analyzing these genomic records of DSBR will shed light on the processes of DSBR and chromosome evolution.

We examined mitochondrial sequences that were inserted into the nuclear genomes of human and chimpanzee after the divergence of the two species about 5–6 Myr ago [16],[18]. Nuclear sequences of mitochondrial origin [*numts*, 19] have been identified in numerous sites throughout nuclear genomes [20]–[22] in species ranging from yeast to plants and humans [23],[24] based on their sequence similarity to mitochondrial DNA. *Numts* are randomly distributed among the chromosomes with no apparent integration hotspots [18],[25]. However, it was suggested that *numt* are common in introns and near repeats [16],[26]. In humans, *numt* sizes range from tens of bases to an entire mitochondrion (16 kb) and represent 430 kb of the genome [18],[25]. *Numts* appear on all chromosomes and integration of *numts* into genes has also been associated with diseases [27],[28]. A deluge of mitochondrial DNA has been steadily transferred to the nucleus since the origin of the mitochondria from the α-proteobacterial endosymbiotic ancestor [29]. All mammalian *numts* studied to date are considered “dead-on-arrival” pseudogenes [30], but evidence for functional *numts* has been reported in species including plants, yeasts and flies [31],[32].

*Numts* have unique characteristics that make them especially suitable to the study of evolutionary signatures of DSBR: i) they are unable to actively integrate into the genome (in contrast to LINEs and SINEs [33]), and instead, are captured into the nuclear genome via an NHEJ mechanism [15]–[17],[34]; ii) they possess no intrinsic ability to transpose after insertion by NHEJ, and thus are a stable marker of a repaired DSB; and iii) since *numts* are derived from the mitochondrial genome, they are easily identified and distinguished from the nuclear DNA by sequence analysis. Thus, the *numt*-chromosome borders are well-defined and sites of fusion (fusion points) can be determined with single-nucleotide resolution.

We describe a comprehensive molecular analysis of *numt* fusion points throughout the human and chimpanzee genomes. Some of the repair patterns observed in experimental systems are detected during chromosome evolution in various genetic loci and natural DSB configurations. Surprisingly, *numt*-mediated NHEJ involves fewer deletions in comparison with experimental NHEJ, whether different types of filler DNA were present or not. We suggest a model according in which filler DNAs may play a role in protecting genome integrity from deleterious processing of DSBs.

## Results

### Comprehensive Analysis of *numt* Integration through Hominoid Evolution Supports NHEJ-Mediated Insertion Mechanism

**Figure 1:**
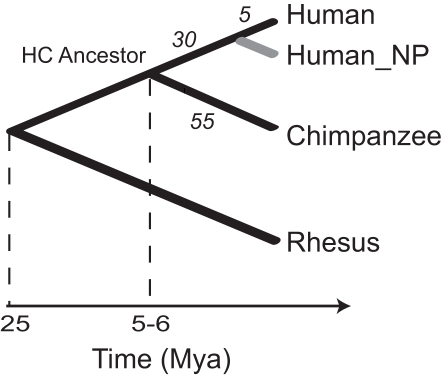
Phylogenetic tree of human, chimpanzee and rhesus monkey showing new *numt* insertions used for DSBR analysis. Recent human and chimpanzee *numt* insertions according to triangulate classification are shown on each branch. The *numt* polymorphic variants [16] (NP, in gray) are optional in our analysis. Human and chimpanzee (HC) common ancestor is indicated.

Human- and chimpanzee-specific *numts* were identified based on the genome alignment of human and chimpanzee using rhesus monkey as an outgroup (Figure 1). In total, 55 chimpanzee-specific *numts* and 35 human-specific *numts* were identified (see Methods).

Two possibilities pertaining to the origin of *numt* previously were considered for the entire *numt* repertoire in the human genome: independent insertion from the mitochondria and genomic duplication subsequent to the insertion [20],[21]. Recent *numts*, however, generally are considered to have been inserted independently from the mitochondria via NHEJ based on experimental studies, lack of homologies in the flanking regions, and the appearance of NHEJ hallmarks in the fusion points [15]–[17],[34]. We tested this hypothesis in this study. *Numts* inserted from the mitochondria potentially could be inserted by homologous recombination between chromosomal and mitochondrial DNA. If this is true, *numt*-flanking regions should show sufficient sequence identity with mitochondrial DNA. To test this possibility, each *numt* identified in the study, along with 100 bases of up- and down-stream flanking chromosomal sequences, was BLASTed against the mitochondria (see Methods). We could not find sequence identities between mitochondria and chromosomal DNA that include more than seven bp from the *numt*, thus, no evidence was found for insertion by homologous recombination.

*Numts* have no self-replicating mechanism, therefore, the prediction is that *numt* duplication is expected to be part of a larger segmental duplication [20],[35],[36]. In this case, *numt* is predicted to insert through non-allelic homologous recombination between chromosomal DNA and preexisting nuclear *numt* and should be characterized by DNA sequence homology that extends beyond the *numt* [20]. To test this possibility, each *numt* identified in the study, along with 100 bases of up- and down-stream flanking chromosomal sequence, was BLASTed against the nuclear genome. None of the *numts* and flanking regions showed sequence identity with the genomic target to account for non-allelic homologous recombination (see Methods). We also looked for *numts* that overlapped with human and chimpanzee segmental duplication [36]. These are genomic duplications characterized by >1 kb and >90% identity. Four out of 90 *numts* showed overlap with segmental duplications. In the cases where *numts* overlap duplicated segments, *numts* were found in only one of the copies while missing from the others, which demonstrated that *numts* were inserted subsequent to the duplication events. Therefore, recent *numts* described in this study cannot be explained by non-allelic homologous recombination.

A *numt* duplication mechanism that is independent of homology was the next possibility that was considered. Promiscuous DNA template switching is the only copying mechanism that was reported to cause genome structural variations in a homology-independent manner [37]–[39]. According to this mechanism, a stalled replication fork invades a nearby template at another DNA replication fork and copies the information available at that locus. The meaning of this mechanism while considering *numt* insertion is that the fork should switch to an alternative template that includes a preexisting *numt*, copy the *numt* and switch back to the original template in a position that is continuous to the first one. This is very unlikely. Nevertheless, the possibility that species-specific *numts* may arise by this mechanism was tested. If *numt*s emerge by promiscuous DNA template switching, one should see additional *numt* copies in the genome of at least the same size, which can act as templates. In addition, if a *numt* has arisen by template switching, it should be phylogenetically more closely related to the donor *numt* than to the mitochondria. Based on DNA distance analysis (see Methods) only 18 out of 90 *numts* showed lower DNA distances to another nuclear sequence than to the mitochondrial one. This number is an upper estimation for *numts* that may have potentially arisen through homologous-independent duplication, since independently arising *numts* can be closely related to one another if they arose in the temporal proximity [20]. Therefore, these 18 *numts* were analyzed further. DNA template switching occurs *in cis*, namely within the same chromosome [38]–[40], which is due to the need to invade an adjacent replication fork [40] and in primates due to chromosome territory [41]. Out of the 18 suspected *numts*, only one could have potentially arisen from a preexisting *numt* on the same chromosome. In addition, none of the *numts* has additional DNA fragments from a donor site, as is expected from template switching mechanism. Finally, promiscuous DNA template switching involves considerable rearrangements of the chromosome, usually with more than a single break point and covers long genomic regions (0.2–7 Mb [38]). *Numt* insertions, on the other hand, are short (0.03–6 Kb) and show a simple and local insertion pattern. Taken together, in our study we conclude that the most parsimonious explanation for species-specific *numt* integration is NHEJ.

**Figure 2:**
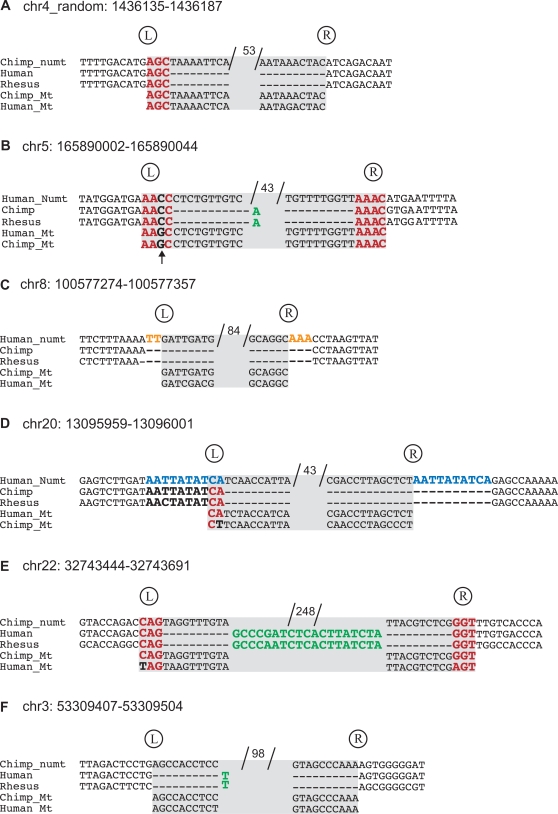
Common forms of *numt*-mediated NHEJ. Each alignment includes a human or chimpanzee locus with a *numt* as well as the corresponding nuclear sequence in the sister taxon and rhesus sequences. The mitochondrial sequences from human and chimpanzee are also indicated. (A) *Numt* insertion involved microhomology of AGC at the left fusion point (shown in red) and blunt-end repair at the right fusion point [id = 97]. (B) The *numt* locus involved imperfect microhomology at the left fusion point and continuous microhomology at the right fusion point. The position marked with an arrow has C in the nuclear genomes but G in the mitochondrial genomes. One base was deleted (green) [id = 16]. (C) *Numt* insertion involved a blunt-end repair with non-templated insertions of two bases at the left fusion point and three bases at the right fusion point (shown in yellow) [id = 19]. (D) *Numt* insertion involved microhomology of two bases on one side (shown in red on left, but could also be on the right) and a chromosomal target sequence duplication of ten nucleotides (blue) on the other side [id = 32]. (E) *Numt* insertion involved microhomology-dependent repair at both fusion points (red). Nineteen bases that appeared in the corresponding locus in the human and rhesus monkey genomes were deleted from one or both of the fusion points [id = 74]. (F) *Numt* insertion was mediated by a blunt-end repair event. One base was deleted (green) [id = 44]. The *numt* region is shown in a gray box. Deletions (in green) are shown in the gray box. *Numts* are trimmed and their size is indicated.

Human and chimpanzee *numt* loci can be used to deduce the mechanism of NHEJ. The hominoid lacking a particular *numt* carries the sequence prior to DNA repair, while the hominoid carrying that *numt* has the sequence reflecting the DSBR event. Thus, the differences between the chromosomal *numt* loci sequences of these two hominoids can be considered to reflect directly NHEJ processing (Figure S1 and Figure 2). Similar argument was previously used for analysis of few human polymorphic *numts* where individuals differ in the presence of a *numt* in specific loci [16]. Note that this inference is possible because human and chimpanzee share a recent common ancestor (Figure 1) with a low mean single-nucleotide substitution rate between their genomes of 1.23% [42].

At each end of a *numt*, there is a junction with chromosomal DNA to one side and mitochondrial DNA on the other, and these junctions reflect the repair events at each end of the original chromosomal break (left or right in Figure 2). Repair of the chromosomal sides of the fusion point can be studied by examining the sequences at the junction, but the donor mitochondrial DNA used to patch the chromosomal DSB cannot be studied since it is no longer present.

To assess repair at the *numt* integration sites, *numts* were examined for deletions in the flanking chromosomal DNA and for evidence of microhomology- versus blunt-directed repair. All 90 species-specific *numts* were tested for evidence of deletions. Of 180 fusion points (90×2 fusion points/*numt*), 152 were analyzed further for microhomology and blunt-end repair (Table 1). Twenty-eight fusion points were excluded from the microhomology and blunt-end repair analysis due to uncertainty in classification (see below and Methods). All *numts* as well as their classifications are shown in Table S1.

**Table 1:** 90 human and chimpanzee *numts* appear in this study and their classification to *numt-*chromosome fusion point. *Numts* are shown according to their two-side classification.

Repair type in left and right fusion points	*Numt* data		Fusion point data			
	*Numts*	*Numts* with deletions	Analyzed fusion-points	Fusion points with microhomology (≥2 bases)	Fusion points with blunt-end repair	Fusion points with blunt-end repair and insertion
Microhomology×Microhomology	8	7	16	16	0	0
Blunt×Blunt	30	7	60	0	45	15 (1 two sides)
Microhomology×Blunt	33	14	66	33	23	10
Cases involved two events (insertion>5) in one fusion point	10	6	10	4	5	1
Events with uncertain classification	9	7	0	ND	ND	ND
**Total**	**90**	**41**	**152**	**53**	**73**	**26**

For a detailed description of each fusion point, see Table S1.

### Frequency of Repair Involving Microhomologies Supports Cassical NHEJ during *numt* Integrations

Microhomology in chromosomal NHEJ has been observed in numerous cases [2],[43],[44]. Some reports have shown that repair that involves microhomology is a statistically significant mechanism of NHEJ, but a single base microhomology is not [12],[45]. While classical NHEJ is very effective even in the absence of microhomology [3],[44], alternative end joining pathways rely almost exclusively on microhomology-related repair [43],[46]. A dataset of 152 junctions was used to study the role of repair involving microhomologies in *numt* integration.

End-joining involving use of microhomology is inferred at the fusion point when both human and chimpanzee chromosomal nuclear sequences overlap with the mitochondrial sequence (e.g., Figure 2A, left side; Figure S2). Eighty-four fusion points involved microhomology of 1–7 bp, in agreement with experimental data [2],[44],[45].

**Figure 3:**
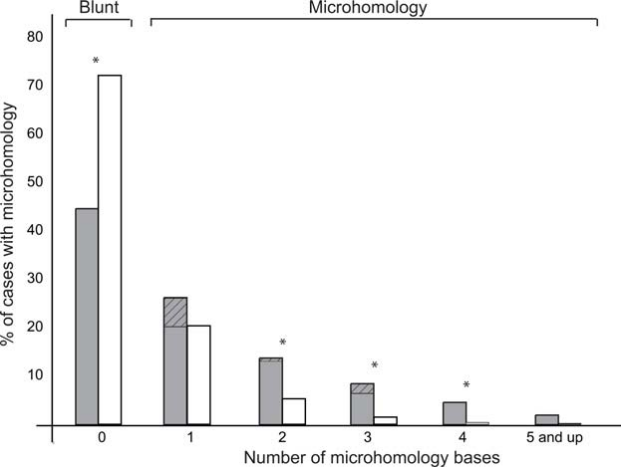
Statistical analysis of DSBR involving microhomologies. The percentages of *numts* (grey) and random (white) datasets with a specific microhomology length are shown. The *numt* dataset includes 152 genome-*numt* fusion points, and the random dataset includes 15,200 randomly chosen genome and mitochondrial positions. A one-tail Fisher exact test (presence/absence of microhomology, *P* = 1.3×10^−12^) was statistically significant. A star indicates a significant difference between *numt* and random datasets for a given microhomology length (binomial test, *P*<0.05/6). The percentage of imperfect microhomology is shown at the top of the graph with a diagonal pattern. Imperfect microhomology was higher in the *numt* dataset than in the random dataset (Fisher exact test, all *P* = 0.01, microhomology of 1, *P* = 0.0023).

The null hypothesis that microhomology is not involved in *numt* insertion, but appears by chance at the point of integration between nuclear and mitochondrial DNAs was tested. This was done by examining 15,200 fusion points of random computer-generated blunt end breaks within the human and mitochondrial genomes (Figure 3). The null hypothesis was rejected (one-tail Fisher exact test, *P* = 1.3×10^−12^), thus, microhomology is involved in the insertion of *numts*. A single base is frequently considered sufficient length for repair mediated by microhomology [6],[47],[48]. According to our analysis, microhomology of a single base was not statistically different from a random single base microhomology (binomial test, n = 3138, p = 1/101, k = 40, *P* = 0.064).

Imperfect microhomology, *i.e.*, microhomology with a mismatch, was considered (Figure 2B). One mismatch was allowed per microhomology stretch, and the base that is adjacent to the fusion point must show microhomology. Only cases where a mismatch in the microhomology stretch increased the stretch by at least two bases were counted. We found that imperfect microhomology is more common in the real *numt* dataset then in the random one (one-tail Fisher exact test, *P* = 0.01). This was highlighted in the case of single microhomologies (one-tail Fisher exact test, *P* = 0.0024). Therefore, repair events involving a single base microhomology were treated as blunt-end repair events, except for those cases that show imperfect microhomology, which were treated as microhomology events. Using this revised definition, 53 (35%) cases of *numt*-genome fusion points employed significant microhomology in the DSBR event (Table 1, see Table S2 for consideration of single base microhomology as microhomology). Thus, it appears that repair involving microhomology plays some role in *numt* integration but is not totally required, as is the case in classical NHEJ. Thus, our extensive dataset provides evidence that repair involving microhomology during NHEJ that was experimentally focused on specific break configuration and genomic locations is a genome-wide phenomenon.

### Target Sequence Duplication during Blunt-End Repair Reveals the Structure of Native Breaks

Ninty-nine repair events (65%) either did not involve any microhomology, or involved only one base microhomology and were considered to be blunt-end repair events (Table 1; Figure 2A right; Figure S2). The processing of *numt*-chromosome blunt fusion points was analyzed. The focus of this section is events that were processed by nucleotide insertions. Nucleotide deletions will be discussed in the next section. In 26 of the 99 blunt-end repair events, nucleotide additions were identified in addition to the *numt* insertion. Nine of these involved insertion of nucleotides that could not be explained by synthesis using a DNA template (Figure 2C). DNA polymerases that act in DNA template-independent manner tend to insert only few nucleotides [49]–[51]. Therefore, we arbitrarily limited the insertion repair events to addition of ≤5 nucleotides (Table 1). Ten larger insertion events were removed from fusion point classification as the *numt* integration likely followed another DNA capture event (Figure S1; Table 1).

In 17 of 71 *numt* integration events (24%) a second type of nucleotide insertion during *numt* integration was observed, where duplication of short chromosomal sequences flanking *numt* fragment was detected (Figure 2D right side). Duplication length ranged from one to 11 nucleotides. These target sequence duplications can occur when 5′ or 3′ cohesive breaks are separated by the mitochondrial DNA following single stranded DNA gap filling during the repair. While the mechanism of integration is different, duplication of the target sequence has been shown for transposable elements such as LINEs [52]. Since *numts* do not induce breaks, but are passively captured into preexisting breaks, we argue that target duplicated sequences represent overhang ends of naturally occurring DSBs. To the best of our knowledge, this is the most comprehensive description of the configuration of naturally occurring DSBs.

Two important insights are drawn from this analysis. First, 24% of integration events were associated with cohesive ends. We suggest that cohesive breaks are probably more frequent in the genome than observed here since those breaks need no processing and are likely to be repaired accurately without patching by *numts*. Second, *numts* inserted in breaks with up to 11 nucleotide overhangs were detected, suggesting that staggered SSBs spaced by 11 nucleotides can lead to a DSB. Since DSBs are more dangerous to genome stability than SSBs it is important to know how close two SSBs on opposite strands should be to be considered a DSB [53].

### *Numt* Insertions Involve Reduced Loss of Chromosomal Nucleotides

**Figure 4:**
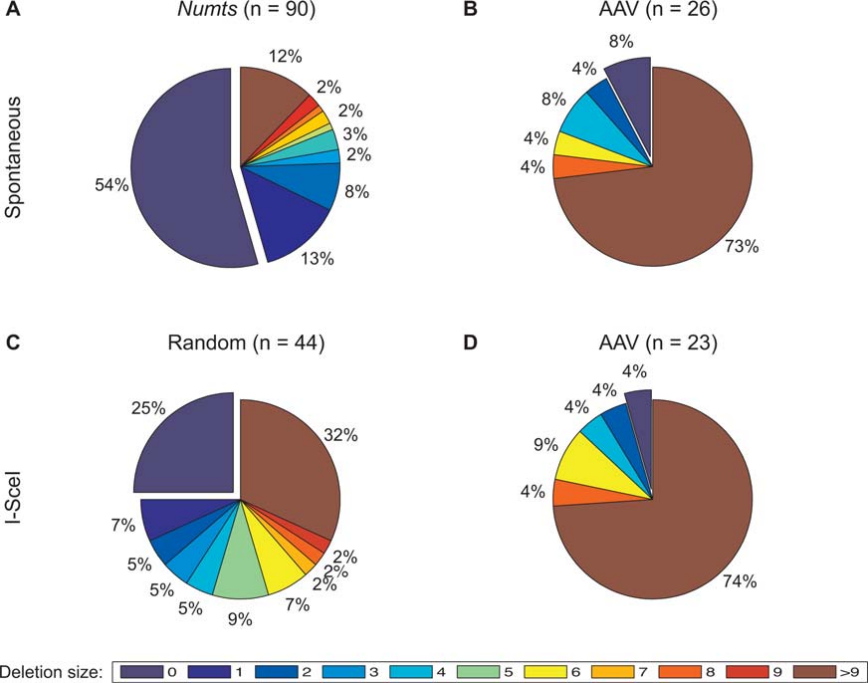
*Numt* insertions involve reduced loss of chromosomal nucleotides. Pie diagrams show the distribution of deletion sizes in our study and different experimental systems with filler DNA (A) *numt*-mediated repair usually involves either no deletions or small 1–2 base deletions, (B) Spontaneous integration of AAV [56]–[58] (C) Random integration into I-SceI [12],[13],[55], and (D) Integration of AAV into I-SceI [56] include more deletions. One-tail Fisher exact tests (presence/absence of deletions, *numt* versus spontaneous AAV *P* = 9.5×10^−6^, *numt* versus AAV *P* = 4.7×10^−6^, and *numt* versus random *P* = 0.001) were statistically significant. Similarly, the meanrank deletion size in the *numt* group is significantly smaller than in the other groups (Kruskal–Wallis nonparametric ANOVA n = 183, *P* = 10^−13^; *A posteriori* Tukey-Kramer test *P* = 0.01). Scale is 0–9 bases, and deletions bigger than 9. Offset slice shows no deletions. A detailed list of deletion size for all non-*numt* events appears in Table S3.

The fusion of mitochondrial and chromosomal DNA likely occurs between incompatible ends, i.e., non-annealing overhangs. In NHEJ studies where filler DNA is not involved, DSBR of incompatible ends always involves deletion of a few nucleotides [3],[5],[54]. For example, in 96 events of Vκ-Jκ recombination, the most studied system for incompatible ends, only one event did not involve deletions [3],[54]. Similarly, in a study of incompatible I-SceI cleavage repair, none of 59 clones preserved the 4-nucleuotide overhangs from the sides of the break [5]. Surprisingly, of the 90 *numt* insertions (Table 1) only 41 (46%) involved deletions of nucleotides from the chromosome (e.g., Figure 2B, E, F). The other 49 insertions (54%) did not involve deletions of even a single nucleotide (Figure 4A). Unlike endonuclease generated breaks, random breaks often involve chemical degradation of the bases at the breakpoint prior to repair [53]. Out of 41 *numt* insertion events that involved deletions, 12 were deletions of a single base, which could have occurred prior to repair. Thus, the frequency of deletions caused by the integration of *numts* could be lower than the observed deletion frequency.

In *numt*-mediated NHEJ mitochondrial DNA acts as filler DNA that may provide the repair machinery an alternative to nuclease activity during chromosome processing. Therefore, this evolutionary study was compared with studies where filler DNA was captured into mammalian chromosomes experimentally [12], [13], [55]–[58]. The filler DNA types that were included in this comparison were spontaneous integration of Adeno-Associated Virus (AAV) into chromosomes, integration of AAV into I-SceI induced breaks and integration of other types of filler DNA into I-SceI sites ([12], [13], [55]–[58], Figure 4). We tested the null hypothesis that the proportion of cases that did not involve deletions would be similar in the *numt* and the experimental filler DNA systems. Spontaneous integration of AAV into genomes involved deletions in the vast majority of cases (24/26, 92% [56]–[58], see Figure 4B). A similar picture was obtained from integration of AAV into I-SceI sites, with 22/23 events (96%) involving deletions ([56], Figure 4D). This effect is not limited to AAV. When other types of DNA [12],[13],[55] (e.g., pCMV and φX174) are captured to I-SceI induced breaks 33/44 events (75%) involve deletions ([12],[13],[55], Figure 4C). We found that the null hypothesis can be rejected and that *numt*-mediated NHEJ involves more events without deletions than experimental systems regardless of the filler DNA used (one-tail Fisher exact tests: *numt* verses spontaneous AAV *P* = 9.5×10^−6^, *numt* verses AAV *P* = 4.7×10^−6^, and *numt* versus random *P* = 0.001).

Moreover, *numts* show a higher frequency of very small deletions (1–2 bases) in comparison to the other three filler DNA studies and a lower frequency of larger deletions (Figure 4). In a comparison of the overall deletion size between repairs involving *numts* versus other filler DNA, the deletion size in *numt* repair events was significantly smaller than the deletion size in all of the other filler DNA groups (Kruskal–Wallis nonparametric ANOVA, n = 183, *P* = 10^−13^, mean ranks of 65.5, 142.5, 99.3 and 128.7 for *numts*, spontaneous AAV, random filler DNA, and I-SceI AAV reciprocally. *A posteriori* Tukey-Kramer test *P* = 0.01). In conclusion, *numts* show less frequent and smaller deletions in comparison to the other three types of filler DNA.

All *numts* described here are inserted into non-coding DNA. Both deletions and insertions are very common during primate evolution [59],[60]. Hence, there is no reason to assume that *numt* insertion events that do not involve deletions are favored over ones that do involve deletions of few bases. Therefore, the low frequency of chromosomal deletions during *numt* insertion is likely to stem from a mechanistic feature of insertion rather then post-insertion selection.

### Mechanism of Deletion Prevention Performed by *numt*s

To understand the mechanisms responsible for reduced chromosomal deletion during *numt* integration we went back to our DSBR analysis (Table 1). In 71 cases where we could analyze the two sides of the integration events (Table 1), 30 showed no microhomology, 33 showed microhomology only in one side, and only 8 showed microhomology on both sides. This suggests that the two sides of DSBs repaired by *numt* insertion are seen independently by repair machinery at least in regard to microhomology- and blunt-end-directed repair.

Interestingly, *numt* insertion via blunt-end repair on both sides of the insertion resulted in the lowest proportion of deletions (7/30). Events involving *numt* insertion via blunt-end repair on one side and microhomology on the other also resulted in a low proportion of deletions (14/33). In sharp contrast, events where both sides of the *numt* were patched through repair involving microhomologies almost always involved deletions (7/8). We conclude that the low deletion rate is not due to selection of *numts* that mimic the complementary strand of chromosomal overhangs, but rather due to the ability to perform blunt-end ligation without trimming chromosomal ends.

## Discussion

Primate genomes are useful for studying the repair of naturally occurring DSBs via *numt* integration because of their high sequence similarity and the availability of multiple complete genomes. While our analysis identified similarities between the pattern of *numt* integration in primate evolution and experimental NHEJ repair patterns (see below), it also highlighted a clear difference between them.

In repair events of incompatible ends, small deletions always occur [3],[5],[6]. Deletions during repair are also common in repair involving filler DNA (Figure 4). While only reports which include raw data are shown in Figure 4, recently Miller *et al.* reported that about 80% of 212 random integration events of AAV into human genome involved deletions larger than 10 bases, and about 30% of the 212 events involving deletions larger than 100 bases from the host chromosome [61].

**Figure 5:**
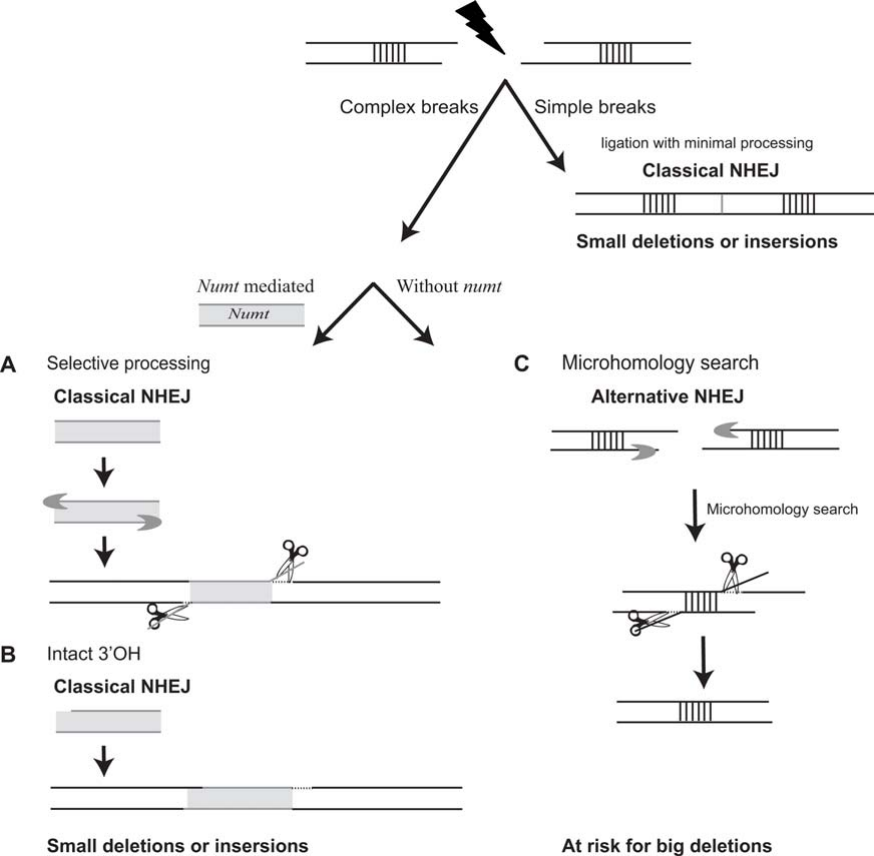
Sealing DSBs with *numts* reduces the risk of deleterious DSBR–a model for protective filler DNAs. DSBs can vary in their chemical complexity, simple breaks are breaks in which the phosphoester bond is broken but neither the sugar nor the nucleotides in the vicinity of the breaks are damaged. In contrast, complex breaks include fragmented sugar and damaged or missing bases in the vicinity of the break. While simple DSBs like experimentally induced ones are repaired with minimal processing, repair of complex DSBs can sometimes be deleterious. We propose that *numts* can seal complex DSBs and thus reduce the risk for deleterious repair. At least two mechanisms may explain how *numts*, and probably other filler DNA, prevent chromosomal deletions. (A) *Numts* prevent deletions if they undergo selective processing that enables them to seal the broken chromosome. For example, if exposure of long single-strand DNA occurs only in the *numt* sequence, *numt*-genome repair mediated by microhomology (dots) can be accomplished even if very short chromosomal single-stranded DNA is exposed at the immediate vicinity of the break. Thus, only non-matching mitochondrial DNA will be removed. (B) Alternatively, some breaks may contain degraded 3′OH in both sides. *Numts* can supply the intact 3′ OH to perform ligation with no need for nuclease processing. (C) In the absence of filler DNA (i.e., *numts*), repair of complex DSBs may not occur in the immediate vicinity of DSB, but instead single-stranded DNA is exposed followed by a search for microhomology between the two sides of the breaks (marked here in strips). When microhomology is found, the two sides of the breaks are annealed, and non-complementary single-strand DNA is trimmed off (scissors) to yield a deletion in the chromosome. Dotted lines represent DNA synthesis and crescent-shaped blobs represent endonucleases.

In contrast, only 46% of *numt* integration events included deletions, and those deletions were small. The differences between the frequency and size of deletion events in *numt*-mediated DSBR and other filler DNA mediated DSBR are statistically significant. Since selection is likely to act similarly on loci with no deletions or only a few base deletions in non-coding regions, the fact that so many *numt* integration events do not involve deletions at all, suggests it is determined by the mechanism of insertion and not by post-insertion selection. Our analysis indicates that *numts* prevent chromosomal deletions primarily through blunt-end repair. At least two mechanisms may explain how *numts* prevent chromosomal deletions. It could be that trimming of DNA ends occurs specifically in the mitochondrial DNA during DSBR (Figure 5A). Alternatively, or in addition, some breaks may contain degraded 3′OH on both sides such that direct ligation or ligation facilitated by DNA synthesis cannot occur. In this situation, the only way to perform end joining is by deleting bases. *Numts* can supply intact 3′ OH for ligation with no need for nuclease processing (Figure 5B).

Although *numt* integration events show a lower frequency of deletion than V(D)J recombination their repair patterns are similar. The frequency of repair involving microhomology is similar in both cases as well as the limited processing of the chromosomal DNA. Therefore, it is reasonable to suggest that *numts* are inserted using classical Ku-dependent, DNA ligase IV-dependent NHEJ [5],[43],[62], and that the information from experimental systems holds true for broader genetic loci and end configurations.

In a broader context *numts* can serve as a model for other types of filler DNA which are frequently found both in evolution and in experimental DSBR. We propose a model in which some types of filler DNA may have a role in protecting chromosomes from deleterious deletions during DSBR (Figure 5). DSBs can vary in their chemical complexity. Simple breaks are breaks in which the phosphoester bond is broken but neither the sugar nor the nucleotides in the vicinity of the breaks are damaged [63]. In contrast, complex breaks include fragmented sugar damaged or missing bases in the vicinity of the break [64],[65]. While simple DSBs generated by endonucleases mainly cause small deletions, it is clear that a subset of random breaks leads to large deletions and translocations [43], [66]–[68]. For example, ionizing radiation induced *hprt* mutants resulted in deletions up to 56 kb whereas improper IgH class switching and spontaneous DSBs can lead to translocations [43], [66]–[68]. Molecular characterization of large deletions and translocations revealed that they are mainly caused by microhomology-mediated repair [8],[43],[67],[69]. Genetic analysis showed that these types of deletions and translocations are probably formed by alternative end-joining [5],[43],[62], where DSBs are processed by nucleases to generate long stretches of single stranded DNA, followed by a microhomology search. When microhomology is found, the two sides of the breaks are annealed, and non-matched single-strand DNA is trimmed off to yield a deletion (Figure 5C). Our results are consistent with a model in which filler DNA may seal breaks and prevent their processing into long single stranded DNA (Figure 5A, B).

Deletions are reduced during NHEJ by restricting the activity of nucleases primarily by the Ku heterodimer [5]. Nuclease activity is limited further during V(D)J recombination by Rag1/2. Deletions also are reduced during V(D)J recombination by DNA polymerase μ [54],[62]. Our results suggest that filler DNA such as *numt* can act similarly to reduce deletion size during the repair of random breaks. *Numts* might carry this out by increasing the efficiency of repair processes that do not involve nuclease activity, for example, by providing an intact 3′OH for DNA polymerases or DNA ligase.

Capture of DNA sequences into chromosomal breaks was shown previously both in cell culture and during chromosomal evolution [12], [13], [55]–[58],[70]. It also was suggested that the captured DNA serves as a repair factor [70]. However, capture of DNA has not been shown to protect the chromosome from deletions, as is indicated here. In fact, as shown in Figure 4 in some experimental designs, capture of DNA into breaks involved frequent and substantial deletions of the chromosome. There are several possible explanations as to why the results obtained with *numts* are different from the results that obtained experimentally (Figure 4).

First, the specific experimental design may influence the repair outcomes. *Numts* are captured into variety of break configurations while DNAs in Figure 4C and D are captured into defined I-SceI breaks. It was shown that incompatible 3′-overhang-breaks that are present during the repair of I-SceI sites with filler DNA are prone to deletions [44]. In addition, during the experiment, the genome undergoes cycles of I-SceI cleavage and end joining until the filler DNA disrupts the I-SceI recognition site. During these cycles, some repair factors can be exhausted that may affect the repair outcomes. Alternatively, the repeated formation of DSBs can signal the cell as if an un-repairable DSB is formed that may lead to a change in the cellular repair strategy [6].

Second, different cell types and developmental stages sometimes can show different repair outcomes [71],[72]. While *numts* in our study are inserted into the germlines *in vivo*, the experiments that are presented in Figure 4 B–D were performed in immortalized cells in culture.

Finally, a very important factor that may explain the differences between *numt*-mediated repair and other filler DNAs is the type of DNA that is captured into the break. It appears that some filler DNA can “protect” from DNA deletions during insertion, while other filler DNA may be promiscuous, promoting alterations. Capture of AAV into both random and I-SceI breaks showed similar results with the highest frequency of deletions (Fig. 4B,D), indicating that in this case the type of DNA has more effect than the type of break. AAV DNA is a single stranded DNA that forms a loop structure at one side and a long single strand tail at the other. Integration sites of AAV to the genome occur in the loop structure [56], therefore the loop structure should be opened up and processed before ligation. During this process, a unique DNA structure of non-complementary base-pairs is formed at the end of the AAV DNA. This structure probably makes blunt end ligation with the chromosome very unlikely to occur. Here, we showed that *numt* integrations prevent deletions mainly through blunt-end repair. It is possible that capture of DNA that cannot be blunt-ligated occurs via a different mechanism that is prone to form deletions. Indeed, the mechanism of insertion of AAV into genomes as suggested by Miller *et al.* [56] is opposite to what we describe here for *numts*. According to their model, AAV integration is promoted by exposure of long ssDNA followed by deletions.

Unlike AAV, the capture of different types of DNA into I-SceI breaks (figure 4C) was more similar to the capture of *numts* with respect to deletion frequency. In addition to the explanations discussed above, in figure 4C different types of sequences are captured into the breaks including capturing of retrotransposon cDNA [12],[13]. Recently, it was shown that retrotransposon cDNA can be captured passively into breaks both in cell cultures and during primate evolution via an NHEJ-independent pathway. This process involves a high frequency of associated deletions (86%) and a large deletion size (up to 14 kb) [70],[73]. Therefore, it can be that the differences that are observed between panels A and C (Figure 4) could be explained partially by the capture of cDNA or other DNA that is integrated similarly.

We speculate that within cells there are others DNA fragments that are captured into DSBs with a similar protective potential as *numts*. Further study of the insertion mechanism of other passively captured filler DNA will identify protective fragments and potentially the rules in their preferential use over promiscuous filler DNA during chromosome evolution. This will add valuable insights into the mechanisms of chromosomal evolution and maintenance of genome integrity.

## Methods

### Identification of Human and Chimpanzee Species-Specific *numts*

Genomic sequences and annotations were obtained from the University of California at Santa Cruz [74] Genome Center. The genome versions used were hg18 (human, *Homo sapiens*), panTro2 (chimpanzee, *Pan troglodytes*), and RheMac2 (rhesus monkey, *Macaca mulatta*). The pair-wise analysis is described in Hazkani-Covo and Graur (2007). In short, BLAST was used to search each of the human and chimpanzee genomes for regions of similarity with mitochondrial sequences. *Numts* were then classified as new based on the alignment files: netPanTro2 for human-chimpanzee, netHg18 for chimpanzee-human, and two netRheMac2 tables for human-rhesus and chimpanzee-rhesus. *Numts* that appeared in one of the two hominoids (human or chimpanzee) and gaps that appeared in the reciprocal locations in the two other genomes were identified. A total of 90 *numts* were identified based on this criteria and are discussed in this study. *Numts* that appeared in one of the two hominoids [18] but could not be compared to the rhesus genome for that loci, were not used in this study.

### DSBR Analysis

First, the possibility that *numt* insertion occurred via homologous recombination between chromosomal and mitochondrial DNA or between chromosomal DNA and nuclear paralogous *numt* was tested. The test was based on the assumption that if such recombination had occurred a significant portion of both the *numt* and its flanking regions should be homologous to a region of the nuclear or mitochondrial genome. To test those options, we BLASTed *numt* including 100 bases of flanking genomic sequence from each side of the fusion point against the nuclear and mitochondrial genomes looking for homologies that cover both the *numts* and the flanking regions. We looked for hits that covered more than 10 bp of each of the flanking region [75].

In order to test overlap of *numts* with segmental duplication, the recent human (Hg18) and chimpanzee (PanTro2) segmental duplications were downloaded from the segmental duplication database [36]. Segmental duplications of fragments that include *numt* regions were tested against the mitochondria using BLAST2SEQUENCES.

To test the option of homologous-independent *numt* duplication, Hasegawa-Kishino-Yano (HKY) DNA distances were calculated using PAUP [76]. Distances were calculated between each *numt* and its mitochondrial fragment and between each *numt* and a possible nuclear template. *Numts* that show lower DNA distances to a nuclear copy rather than to the mitochondria were analyzed.

Loci of species-specific *numts* were aligned with the two other nuclear genomes and with mitochondria from human and chimpanzee. Flanking genomic regions of 100 bp were used from each side of the *numt*. Alignments were created with ClustalW [77] and inspected manually.

*Numts* then were classified for the underling NHEJ patterns (microhomology, blunt end repair, and deletion size). Each side of the *numt* was analyzed independently according to the following parameters. First *microhomology* was indicated only if the nucleotide adjacent to the fusion point was shared between the *numt*, the comparative hominoid genome, and the mitochondrion of human or chimpanzee. The length of microhomology was defined as a set of continuous sites that each obeyed this rule. Available *numt* polymorphic sites were used in the analysis. If no microhomology was observed, then the DSBR was classified as a *blunt-end repair* event. Insertions were classified as a chromosomal target sequence duplication if the insertion was identical to the nucleotide(s) appearing in a genomic sequence on the other side of the *numt*. All other insertions were classified as non-template insertions. Where insertion of more than five nucleotides in a non-templated manner was observed in addition to the *numt* insertion, the fusion-point was removed from the study (Table 1). Genomic deletion events were counted per *numt* rather than per fusion point. Deletions were detected based on comparisons between genomes with and without a *numt* at a particular location. While *numts* with poorly aligned flanking regions were not included in the fusion point classification due to uncertainty (Table 1), estimates of the size of deletion was reported. Overestimation of deletion was determined in case of doubt.

## Supporting Information

Figure S1 Chimpanzee genome is used to identify *numt*-genome fusion points of human specific *numts*. Five human genomic loci with *numt* polymorphisms are shown. Each alignment includes two human variants (with and without the *numt*) as well as the corresponding chimpanzee and rhesus sequences. The *numt* region is shown in a gray box, and the conservation line between human and chimpanzee is presented. (A–C) Cases where the sequences around *numt* integration site are identical between the *numt* polymorphic (NP) human variant and that of chimpanzee. (D) An independent tandem duplication of CAAA occurred in the lineage leading to human. (E) The comparative genome approach is not always useful. At the right side of the *numt*-genome fusion point, an insertion of fourteen bases is observed in addition to the *numt* insertion. The event that occurred at the left *numt*-genome fusion point is not clear since the AGG triplet can be aligned on both sides of the gap that was formed in the alignment. Cases like E were not included in the fusion point analysis. The following refSNP ID and study case numbers were used: rs35075891, id = 1; rs35867387, id = 3; rs1052523, id = 22; rs11271404, id = 6; and rs34351771, id = 31.
(3.54 MB EPS)

Figure S2 Repair of a DSB by *numt*-mediated NHEJ using microhomology and blunt-end repair sub-mechanisms. (A) A non-cohesive putative DSB is created in the chromosome strands with a blunt-end on the left side and a protruding end of 2 bases on the right side. The DSB is then repaired by a putative mitochondrial piece (gray) yielding a blunt-end repair reaction on the left and a microhomology-mediated reaction with two bases on the right. (B) The alignment of the repaired DNA in A (upper sequence) with the corresponding loci in two closely related species is shown. Note that on the right fusion-point where microhomology-mediated repair happened an overlap of two bases appears between the two intact sequences and the mitochondrial sequence (grey).
(0.47 MB EPS)

Table S1 Detailed classification of *numts* that are analyzed in this study. For each of the 90 *numts*, information about chromosome and mitochondria coordinates as well as the DSBR pattern for left and right fusion points are indicated. Blunt-end repair/microhomology-mediated repair as well as non-template insertions are indicated for left and right separately. In case of microhomology the actual bases are given. In addition, scores of microhomology for 8 bases upstream of the left fusion point (and downstream of the right one) are shown in a binary way, where 1 indicates a match and 0 a mismatch. For example, the alignment of CAG appear in Figure 3d (id = 74) is 00100111 (The binary code is decoded from right to left as match-match-match (CAG) mismatch-mismatch-match-mismatch-mismatch). Note that octamer orientation is the same as the sequence orientation and is opposite for left and for right fusion points. Cases where non-continuous microhomology was determinate are indicated in the last column (e.g., id = 99, L-3p2 means in the left side 3 bases of microhomology can be extended by two additional bases when allowing a mismatch). Template insertions as well as deletions are indicated per *numt* rather than per fusion point. ^a^ *Numt* includes additional insertion of at least 5 bases. This insertion is, therefore, suspected to be a separate event. Only the side without insertion was counted for the DSBR patterns. ^b^ Classification of *numt* to its repair pattern was ambiguous, therefore only deletion size was estimated.
(0.06 MB XLS)

Table S2 90 human and chimpanzee *numts* appear in this study and their classification to *numt*-chromosome fusion point. Microhomology of a single base is classified here as microhomology in contrast to Table 1 where a single base microhomology is considered as blunt-end repair. *Numts* are shown according to their two-side classification. For a detailed description of each fusion point, see Table S1.
(0.05 MB DOC)

Table S3 Deletion size in cases of NHEJ with filler DNA. For deletion size of *numt*-mediated repair see Table S1.
(0.04 MB DOC)
