# Supplementary material for: Numt-Mediated Double-Strand Break Repair Mitigates Deletions during Primate Genome Evolution
Source: PLoS Genet. 2008 Oct 24;4(10):e1000237. doi: 10.1371/journal.pgen.1000237 (PMC2567098; doi:10.1371/journal.pgen.1000237)
Supplement: Table S2 — 90 human and chimpanzee numts appear in this study and their classification to numt-chromosome fusion point. Microhomology of a single base is classified here as microhomology in contrast to Table 1 where a single base microhomology is considered as blunt-end repair. Numts are shown according to their two-side classification. For a detailed description of each fusion point, see Table S1. (0.05 MB DOC) [file pgen.1000237.s004.doc]

|  | *Numt* data | | Fusion point data | | | |
| --- | --- | --- | --- | --- | --- | --- |
| Repair type in left and right fusion points | *Numts* | *Numts* with deletions | Analyzed fusion-points | Fusion points with  microhomology  (≥ 1 bases) | Fusion points with blunt-end repair | Fusion points with blunt-end repair and insertion |
| Microhomology x Microhomology | 19 | 11 | 38 | 38 |  |  |
| Blunt x Blunt | 11 | 3 | 22 | 0 | 15 | 7 (1 two sides) |
| Microhomology x Blunt | 41 | 14 | 82 | 41 | 23 | 18 |
| Cases involved two events (insertion>5) in one fusion point | 10 | 6 | 10 | 5 | 4 | 1 |
| Events with uncertain classification | 9 | 7 | 0 | ND | ND | ND |
| **Total** |  | **41** | **152** | **84** | **42** | **26** |

**Table S2 – 90 human and chimpanzee *numts* appear in this study and their classification to *numt-*chromosome fusion point. Microhomology of a single base is classified here as microhomology in contrast to Table 1 where a single base microhomology is considered as blunt-end repair. *Numts* are shown according to their two-side classification. For a detailed description of each fusion point, see Table S1.**
