# Supplementary material for: Numt-Mediated Double-Strand Break Repair Mitigates Deletions during Primate Genome Evolution
Source: PLoS Genet. 2008 Oct 24;4(10):e1000237. doi: 10.1371/journal.pgen.1000237 (PMC2567098; doi:10.1371/journal.pgen.1000237)
Supplement: Table S3 — Deletion size in cases of NHEJ with filler DNA. For deletion size of numt-mediated repair see Table S1. (0.04 MB DOC) [file pgen.1000237.s005.doc]

**Table S3. Deletion size in cases of NHEJ with filler DNA. For deletion size of *numt*-mediated repair see Table S1.**

| Type of break | Type of filler | Number of integrations analyzed | Deletion size | Reference |
| --- | --- | --- | --- | --- |
| Spontaneous | AAV | 26 | 0, 0, 2, 4, 4, 8, 9, 13, 21, 22, 23, 24, 27, 33, 58, 71, 119, 211, 215, 277, 278, 2107, 87824, 133512, 164875, 359742 | [56] Figure 3d,  [57] Figure 1,  [58] Table 1 (only cases where both fusion point were analyzed included). |
| I-SceI | AAV | 23 | 0, 1, 2, 5, 5, 9, 10, 10, 10, 11, 11, 12, 13, 13, 14, 17, 23, 27, 29, 121, 130, 134, 1966 | [56] Figure 3a-c |
| I-SceI | Random | 44 | 0, 0, 0, 0, 0, 0, 0, 0, 0, 0, 0, 1, 1, 1, 2, 2, 3, 3, 4, 4, 5, 5, 5, 5, 6, 6, 6, 7, 8, 9, 13, 19, 22, 23, 24, 26, 28, 29, 34, 39, 45, 47, 67, 101 | [12] Table2,  [13] Table3,  [55] Figure 6. |
